# Comparisons of medical utilizations and categorical diagnoses of emergency visits between the elderly with catastrophic illness certificates and those without

**DOI:** 10.1186/1472-6963-13-152

**Published:** 2013-04-26

**Authors:** Yang Nan-Ping, Lee Yi-Hui, Chung Chi-Yu, Hsu Jin-Chyr, Yu I-Liang, Chang Nien-Tzu, Chan Chien-Lung

**Affiliations:** 1 Institute of Public Health, College of Medicine, National Yang-Ming University, Taipei, Taiwan; 2 Department of Medical Research, Tao-Yuan General Hospital, Department of Health, Executive Yuan, 1492, Jhongshan Rd, Taoyuan, 33004, Taiwan; 3 Institute & Department of Nursing, College of Medicine, National Taiwan University, Taipei, Taiwan; 4 Department of Nursing, School of Nursing, Chang-Gang University, Taoyuan, Taiwan; 5 Department of Information Management, Yuan-Ze University, Taoyuan, Taiwan

**Keywords:** Elderly, Catastrophic illness, Utilization

## Abstract

**Background:**

In Taiwan, the policy of catastrophic illness certificates has benefited some populations with specific diseases, but its effect on the use of medical services and the sequence of public health has not been examined. As a pilot of a series of studies, focused on emergency department (ED) visits, the present study aimed to compare medical utilization and various diagnostic categories at EDs between the elderly with an identified catastrophic illness and the elderly without.

**Methods:**

A cross-sectional study, based on a large-sample nationwide database (one million of the population, randomly sampled from Taiwan’s National Health Insurance Research Database (NHIRD)), was performed in Taiwan. The 2008 insurance records of ambulatory medical services for subjects aged 65 years or more among the above one million of the population were further selected and analyzed. Taiwan’s registered catastrophic illness dataset for 2008 was linked in order to identify the target subgroup.

**Results:**

The prevalence of certificated catastrophic illness in Taiwan’s elderly utilizing ambulatory medical services was 10.16%. On average, 61.62 emergency department (ED) visits/1,000 persons (95% CI: 59.22–64.01) per month was estimated for the elderly Taiwanese with catastrophic illness, which was significantly greater than that for the elderly without a catastrophic illness (mean 33.53, 95% CI: 32.34–34.71). A significantly greater total medical expenditure for emergency care was observed in the catastrophic illness subgroup (US$145.6 ± 193.5) as compared with the non-catastrophic illness group (US$108.7 ± 338.0) (*p* < 0.001). The three most common medical problems diagnosed when visiting EDs were injury/poisoning (14.22%), genitourinary disorders (11.26%) and neoplasm-related morbidity (10.77%) for the elderly population with a catastrophic illness, which differed from those for the elderly without a catastrophic illness.

**Conclusions:**

In Taiwan, the elderly with any certificated catastrophic illness had significantly more ED visits and a higher ED medical cost due to untypical medical complaints.

## Background

A nationwide health insurance system improves the quality of life and promotes the health of residents, especially when the insurance tax is low, and when many medical facilities are provided. A study evaluating medical utilization based on the Taiwan NHI system in 2002 revealed that on average, a person had 13.4 physician consultations and consulted 3.4 specialties, 5.2 physicians, and 3.9 healthcare facilities in a year; 17.3% of the studied cohort had visited different healthcare facilities on the same day; and 23.5% had visited physicians of the same specialty at different healthcare facilities within 7 days [1]. To decrease inappropriate use and overuse of medical facilities, a co-payment method had been introduced into Taiwan’s healthcare system, which adds an economic load that increases proportionally with the use of medical services. However, this could pose great difficulty for those with specific diseases or those of a low socio-economic level. The policy of listing NHI-defined catastrophic illnesses exempts some vulnerable populations from the co-payment economic burden and protects their human rights with regards to access to necessary medical care. There is some debate regarding equity and adequacy issues, as NHI-defined catastrophic illness may not necessarily cause high medical costs, whereas some expensive diseases or treatments may not be included in such an official list. A study analyzing the correlation between NHI-defined catastrophic illness and high medical expenditure revealed that those with catastrophic illness tended to be socio-economically vulnerable and usually had a high medical expenditure [2].

In Taiwan, the aging society has become an important issue. Owing to progress in the economy as well as in preventive medicine, the life span of Taiwanese people has increased markedly in recent years. This phenomenon has been combined with an unprecedented decline in the overall fertility rate to create a rapidly aging society. As a result, some chronic medical conditions have become major public health problems. Promoting health among the elderly is an important medical issue; in fact, the elderly population has been identified as one of the target populations in the "Healthy People 2020 in Taiwan" program [3]. Therefore, after the 10-year long-term care project was proposed by the Taiwan government, the supply of health care services and the long-term demands for the elderly or those with a catastrophic illness are important for future Taiwanese society [4].

To understand the effect of catastrophic illness certification on the use of medical services and the sequence of public health in Taiwan, a simple descriptive, sampled population-based survey maybe is valuable and can be processed as a pilot of a series of studies. Designed as a cross-sectional, randomly-sampled study, the present study was focused on emergency department (ED) visits and aimed to compare medical utilization and various diagnostic categories at EDs between the elderly with an identified catastrophic illness and the elderly without.

## Methods

### Source, security, and quality control of data

Taiwan launched a single-payer National Health Insurance (NHI) Program on March 1, 1995. As of 2007, 22.60 million of Taiwan’s total population of 22.96 million were enrolled in this program; foreigners in Taiwan are also eligible for inclusion. This universal national health insurance, financed jointly by payroll taxes, subsidies, and individual premiums, commenced in Taiwan, and its coverage expanded from 57% of the population (before the introduction of national health insurance) to more than 98% (after the year 2005). All enrollees enjoy almost free access to healthcare with a small co-payment by most clinics and hospitals [5,6]. In order to respond to current and emerging health issues rapidly and effectively, the National Health Research Institute (NHRI), in cooperation with the National Health Insurance Bureau (NHIB), established a nationwide research database. The NHIB has established a uniform system to control the quality of medical services and coding. The NHRI safeguards the privacy and confidentiality of those included in the database and routinely transfers health insurance data from the NHIB to enable health researchers to analyze and improve the health of Taiwan’s citizens. The NHI database contains registration files and original claims data for reimbursement, and access to the National Health Insurance Research Database (NHIRD), which was derived from this system by the NHIB and is maintained by the NHRI, is provided to scientists in Taiwan for research purposes [7-9].

### Data protection and permission

Data in the NHIRD that could be used to identify patients or care providers, including medical institutions and physicians, is scrambled before being sent to the NHRI for database inclusion, and is further scrambled before being released to each researcher. Theoretically, it is impossible to query the data alone to identify individuals at any level using this database. All researchers who wish to use the NHIRD and its data subsets are required to sign a written agreement declaring that they have no intention of attempting to obtain information that could potentially violate the privacy of patients or care providers. This study protocol was evaluated by the NHRI, who gave their agreement to the planned analysis of the NHIRD. This study was also approved by the Institutional Review Board (IRB) of Taoyuan General Hospital, which has been certificated by the Department of Health, Taiwan (IRB Approval Number: TYGH101044).

### Selection of target population and definition of catastrophic illness

A retrospective fixed cohort population was used as the original study population. Every claimant of the NHI Program at any time during 2005 was included in the population (22,717,053 people) selected for random sampling. Using a random number generator to produce 1,073,891 random numbers, the original claims data of 1,000,000 randomly-sampled beneficiaries from the year 2005 was the original study population of the present study. The majority of the sample population of 1,000,000 subjects were aged from 20 to 65 years, and the male and female subpopulations were of a similar size (49.6% *vs*. 50.4%, respectively). There were no significant differences in the gender distribution, age distribution or average insured payroll-related amount between the claimants in the 2005 sampled data and the original NHIRD [7,8]. The registration and claims data of these 1,000,000 individuals collected by the NHI Program could be followed until 2008. In 2008, all the subjects aged 65 years or older were selected from the original study population to be the sampled population for a further analysis.

If a patient’s ailment is diagnosed by a physician as a “catastrophic illness” under Department of Health guidelines, the patient can submit related information and apply for a catastrophic illness certificate. The application will be formally reviewed, and if approved, the information is entered into his or her IC card [10]. Therefore, the 2008 registered catastrophic illness dataset of Taiwan (including 30 categories, presented in the result section) was used to verify the target cases from the sampled population.

Moreover, emergency cases served by the NHI system are recorded within the dataset of “ambulatory care expenditure by visit”, and all cases classified as a “medical emergency” could be identified. Finally, to study the difference in ED utilization between Taiwan’s elderly with a catastrophic illness and those without, subjects aged 65 years or older in 2008 were selected and their medical records regarding ambulatory services in 2008 were analyzed.

### Statistical analysis

Descriptive statistics are represented as numbers of cases, percentages, and means with standard deviation (SD). The independent *t*-test and 95% confidence interval (95% CI) were used to evaluate the significance of differences between the catastrophic and non-catastrophic illnesses and the psychiatric and non-psychiatric disorders among these catastrophic illnesses. All statistical calculations were performed using the Statistical Package for Social Sciences for Windows (SPSS for Windows 15.0).

## Results

### Basic characteristics of the studied population

Table 1 shows the gender and age-strata distributions of the enrolled subjects, including the elderly with any catastrophic illness and those without in Taiwan based on the national health insurance data for ambulatory care services of one million randomly-sampled beneficiaries in 2008. In general, there were 46,057 males and 50,854 females selected into the present study, and in total 9,842 subjects were identified as having a catastrophic illness. The prevalence of certificated catastrophic illness of Taiwan’s elderly utilizing ambulatory medical services was 10.16% (9,842/96,911). Within these subjects, males were more prevalent than females (10.37% and 9.96%, respectively).

**Table 1:** The enrolled subjects aged 65 years or older selected from a one-million-subject randomly-sampled dataset in Taiwan, 2008

	**Catastrophic illness group**			**Non-catastrophic illness group**			**In general (N = 96,911)**	
Age (years)	Male	Female	Total	Male	Female	Total	Male	Female
65–69	1,173	1,477	2,650	13,296	15,223	28,519	14,469	16,700
70–74	1,212	1,363	2,575	10,148	12,412	22,560	11,360	13,775
75–79	1,161	1,073	2,234	9,043	8,996	18,039	10,204	10,069
80–84	797	692	1,489	5,929	5,669	11,598	6,726	6,361
80 or more	435	459	894	2,863	3,490	6,353	3,298	3,949
Total	4,778	5,064	9,842	41,279	45,790	80,769	46,057	50,854

### Distributions of catastrophic illnesses of the enrolled subjects

According to the Taiwan health authority’s classification, there were 30 categories of catastrophic illnesses and, the studied population comprised 10,217 catastrophic illness cases (Table 2). Among them, the largest category of catastrophic disease was cancer (59.46%), followed by chronic mental disorders (15.39%) and renal failure (9.41%).

**Table 2:** **The distributions of catastrophic illnesses**^**a **^**in the studied subjects based on the official registered system in Taiwan**

**(Category) Disease**	**No.**^**b**^
(1)Cancer	**6,075**
(2&3) Homological abnormality (coagulopathy, anemia)	**15**
Renal failure	**9,61**
(4) Generalized autoimmune diseases	**617**
(6) Chronic mental disorders	**1,572**
(7) Congenital metabolic disorders	**4**
(8) Major organs abnormality	**56**
(9) Massive burns	**9**
(10) Major organs transplantation	**21**
(11) Complicated nervous, musculoskeletal disorders	**5**
(12) Injury severity score more than 16	**125**
(13) Respiratory failure	**277**
(14) Un-corrected mal-nutrition status	**5**
(16) Myasthenia gravis	**31**
(18) Spinal cord injuries	**73**
(19) Occupational diseases	**251**
(20) Cerebro-vascular diseases (acute stage)	**5**
(21) Multiple sclerosis	**1**
(24) Leprosy	**16**
(25) Complicated liver cirrhosis	**87**
(27) Toxic effect of arsenic and its compounds	**6**
(29) Creutzfeldt-Jakob disease	**5**

^**a**^: The other catastrophic illnesses not showed in this tables were: (5) persisted autoimmune disorders, (15) persisted complications due to decompression sickness or air embolism, (17) congenital immune deficiency, (22) congenital muscular dystrophy, (23) congenital anomalies integument, (26) co-morbidity due to premature birth, (28) motor neuron disease and (30) other rare diseases.

^**b**^: total 10,217 cases.

### Comparison of ED utilization between the catastrophic illness and non-catastrophic illness groups

**Figure 1:**
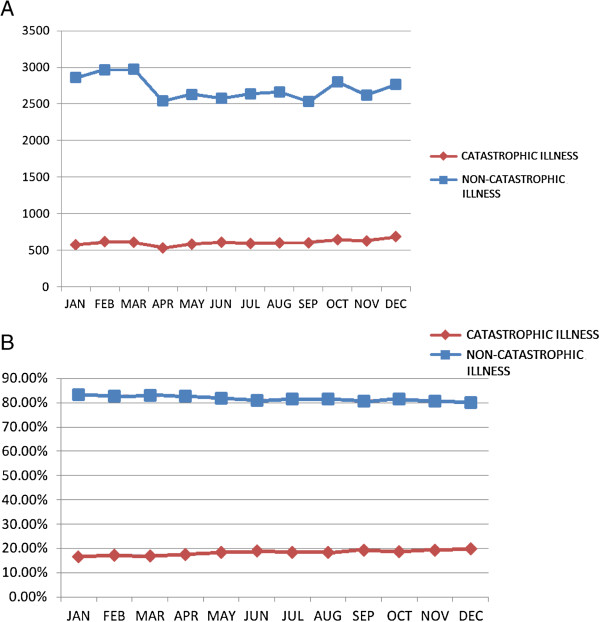
**The distribution of ED visits in various months for the two compared subgroups.** (**A**) A seasonal effect can be seen in the non-catastrophic illness subgroup, as compared with the catastrophic illness subgroup visiting EDs, for which no seasonal effect was observed. (**B**) A higher proportion of ED visits was noted for the catastrophic illness subgroup as compared with the prevalence.

The monthly ED visits of the catastrophic illness group and the non-catastrophic illness group are illustrated in Figure 1A. Compared to the catastrophic illness subgroup, an increased number of ED visits was noted between January and March in the non-catastrophic illness subgroup. In particular, a higher proportion of ED visits (18.30%) was found for the catastrophic illness subgroup compared to the prevalence (10.16%), as shown in Figure 1B. Further analysis must be performed. Table 3 shows a comparison of the utilization of emergency medical services between the two subgroups. On average, 61.62 EDs visits/1,000 persons (95% CI: 59.22–64.01) per month was estimated for the elderly Taiwanese with a catastrophic illness, which was significantly greater than that for the non-catastrophic illness group (average 33.53 EDs visits/1,000 persons per month, with a 95% CI of 32.34–34.71). A significantly greater total medical expenditure on emergency care was observed in the catastrophic illness subgroup (mean NT$4367.2 ± 5804.4, i.e., US$145.6 ± 193.5) as compared with the non-catastrophic illness group (mean NT$3260.6 ± 10138.4, i.e., US$108.7 ± 338.0) (*p* < 0.001). Meanwhile, the majority of the medical expenditure was used for treatment-associated costs (accounting for 78% and 72%, respectively), which differed significantly between the two groups.

**Table 3:** Utilization of emergency medical services in the two compared subgroups

	**Catastrophic illness**		**Non-catastrophic illness**		***p *value**
ED-visited subjects	7,281		32,512		
Averaged ED visits per 1,000 persons monthly (95% CI)	61.62 (59.22 , 64.01)		33.53 (32.34 , 34.71)		
Mean (S.D.) of drug cost per visit ($NT)	324.6	(1334.3)	311.6	(9417.1)	0.779
Mean (S.D.) of treatment cost per visit ($NT)	3402.1	(5279.4)	2336.3	(3426.0)	<0.001
Mean (S.D.) of total medical cost per visit ($NT)	4367.2	(5804.4)	3260.6	(10138.4)	<0.001

95% CI: 95% Confidence Interval.

### Utilization of emergency medical services in the psychiatry-related catastrophic illness subjects and the non-psychiatry-related subjects

Within the elderly with a catastrophic illness, does the utilization of emergency medical services differ between those with a mental disorder and those with a physical disorder? The average number of ED visits was slightly greater for those with a physical catastrophic illness, but this was not significant; however, the total medical expenditure and the treatment-associated cost were significantly higher for the physical catastrophic illness subjects than the psychiatry-related catastrophic illness subjects (*p* < 0.001) (Table 4).

**Table 4:** Utilization of emergency medical services of the subjects with catastrophic illness who had mental-related disorders and those with other disorders

	**Catastrophic illness : psychiatric disorders**		**Catastrophic illness : other disorders**		***p *value**
ED-visited subjects	1,123		6,319		
Averaged ED visits per 1,000 persons monthly (95% CI)	59.37 (56.29 , 62.45)		63.65 (60.63, 66.68)		
Mean (S.D.) of drug cost per visit ($NT)	305.8	(921.2)	350.9	(1391.3)	0.295
Mean (S.D.) of treatment cost per visit ($NT)	2883.4	(3838.8)	3495.2	(5512.5)	< 0.001
Mean (S.D.) of total medical cost per visit ($NT)	3801.8	(4420.7)	4470.7	(6736.1)	< 0.001

95% CI: 95% Confidence Interval.

### Comparisons of diagnostic categories of ED visits between the catastrophic illness and non-catastrophic illness groups

Additionally, the diagnostic categories of the ED visits were compared between the two studied subgroups. With the exception of ill-defined symptoms or signs, the three most definite medical problems when visiting EDs were injury/poisoning (19.43%), circulatory disorders (12.15%) and digestive disorders (9.49%) for the non-catastrophic illness elderly population; these differed from those for the catastrophic illness elderly subjects, which were injury/poisoning (14.22%), genitourinary disorders (11.26%) and neoplasm-related morbidity (10.77%) (Table 5).

**Table 5:** Comparison of diagnostic categories of ED visits between the two compared subgroups

		**Catastrophic illness**		**Non-catastrophic illness**	
ICD-9 codes	Diagnostic category	No.	(%)	No.	(%)
001-139	Infectious and parasitic diseases	96	1.29	406	1.25
140-239	Neoplasms	799	10.77	69	0.21
240-279	Endocrine, nutritional and metabolic diseases and immunity disorders	282	3.80	1,334	4.11
280-289	Diseases of the blood and blood-forming organs	41	0.55	161	0.50
290-319	Mental disorders	161	2.17	323	1.00
320-389	Diseases of the nervous system and sense organs	165	2.22	1,124	3.47
390-459	Diseases of the circulatory system	667	8.99	3,941	12.15
460-519	Diseases of the respiratory system	630	8.49	2,527	7.79
520-579	Diseases of the digestive system	645	8.70	3,079	9.49
580-629	Diseases of the genitourinary system	835	11.26	1,472	4.54
680-709	Diseases of the skin and subcutaneous tissue	125	1.69	733	2.26
710-739	Diseases of the musculoskeletal system and connective tissue	220	2.97	1,177	3.63
780-799	Symptoms, signs, and ill-defined conditions	1696	22.87	9,787	30.18
800-999	Injury and poisoning	1055	14.22	6,301	19.43

## Discussions

In Taiwan, patients with a catastrophic illness certificate who receive care for the illness or related conditions within the certificate’s validity period do not need to pay copayments for outpatient or inpatient care. Catastrophic illness patients must still follow normal treatment and payment procedures when seeking care for unrelated conditions [10]. However, the policy of catastrophic illness certificates, meaning freedom from economic load, indeed benefits these patients with the listed disorders and changes the epidemiological presentation of these diseases in Taiwan. For example, an epidemiologic study of both pediatric and adult systemic lupus erythematosus (SLE) in Taiwan, based on the National Health Insurance Research Database (NHIRD), showed that Taiwan’s incidence and prevalence were higher than those reported in most studies on white populations, and the prevalence increased steadily during the study period, from 42.2/100,000 in 2000 to 67.4/100,000 in 2007 [11]. Another study revealed that the prevalence and incidence of chronic kidney disease (CKD) in Taiwan are relatively high as compared with other countries, and the incidence of end-stage renal disease (ESRD) in Taiwan is the highest in the world [12].

Through analysis of Taiwan’s NHI data, the cumulative prevalence of schizophrenia was found to have increased from 3.34 per 1000 to 6.42 per 1000 from 1996 to 2001, and the annual incidence density decreased from 0.95 per 1000/year to 0.45 per 1000/year from 1997 to 2001. According to the trends of cumulative prevalence and incidence density, the treated prevalence and incidence rate will be approximate to community rates. This means that most people with schizophrenia had received treatment in Taiwan after the NHI program was implemented [13]. Compared with enrollees with a minor psychiatric disorder, those with a major psychiatric disorder have a higher use and a greater cost of mental health care services [14]. Based on the NHI data and the catastrophic illness register data, the co-morbidity of malignancy with other diseases listed as catastrophic illnesses has also been studied in Taiwan [15-17]. Furthermore, many Taiwanese cancer patients could potentially benefit from hospice care; the rate of hospice utilization during their final year of life was calculated, and was shown to have grown substantially from 5.5% to 15.4% from 2000 to 2004 [18].

The implementation of Taiwan's NHI has significantly increased the utilization of both outpatient and inpatient care among the elderly, and such effects are more salient for people in the low- or middle-income groups [19]. A study of 519,003 visits to adult EDs in 12 Taiwanese medical centers sampled in 2000 showed that the elderly accounted for 28.5% of all adult ED visits, and elderly patients accounted for 40.8% of the total adult ED cost. Compared with younger patients, a greater proportion of elderly patients have chronic diseases, are major cases, and are higher-level emergency cases [20]. Different evaluations of the requirement for medical services for the elderly in Taiwan, including the Survey of Health and Living Status of the Elderly in Taiwan (SHLSET) and the National Health Interview Survey in Taiwan (NHIS), have shown that a worsening health status is associated with an increased likelihood of subsequent institutional care use, and the high-comorbidity group tended to utilize more ambulatory care services [21,22].

In the US, a large cohort of persons with SLE who underwent annual structured interviews showed that in those with SLE, a greater disease activity and Medicaid insurance are associated with more frequent ED use [23]. Another study estimated that the average total yearly expense per patient for rheumatoid arthritis increased from 1,155 United States Dollars (USD) in 2000 to 1,821 USD in 2007 [24]. That meant that great physical and economic burdens are suffered by these chronic illness patients, and their medical behavior may be influenced by the conditions of their insurance. In Taiwan, those with any of the listed catastrophic illnesses receive full and free medical care provided by the nationwide healthcare system that could contribute to their family support and the social security, especially for the elderly. For example, a study to examine the effects of chronic kidney disease (CKD) severity and aging on medical utilization in the Taiwanese elderly population revealed that compared with the reference group, increases in medical utilization and expenses were demonstrated in elderly CKD subjects, especially those with late-stage CKD [25].

Based on randomly-sampled cases from Taiwan’s NHI data, the direct medical cost of one ED visit in Taiwan averaged NT$1,792 (US$54.3) for insurers in 2004, and the annual increase of expenditure for emergency medicine was estimated to be 4.9% (*p* < 0.001). In addition, the average treatment-associated expenditure and drug-associated expenditure in Taiwan EDs were 64.5% and 10.6% of the total ED-associated cost, respectively. In particular, treatment-associated cost markedly increased with age (8% per year, *p* < 0.001) [26]. Compared with the present study, it is apparent that the elderly have a much greater medical expenditure at EDs, and, in Taiwan, more than 70% of the total ED medical cost, either for catastrophic illness or not, is treatment-associated, which could possibly be due to the complexity of the medical problems in the elderly.

Otherwise, the frequency of major medical problems diagnosed at ED visits varied by age: the subjects aged 65 years or older had the highest percentage of multiple diagnoses (43%), and the most common diagnostic category among this elderly subgroup was ill-defined symptoms/signs (32.2%), which was noted in 55.9% of cases with multiple diagnoses [26]. In the present study, it was found that the highest proportion for any diagnostic category was about 22% and 30% for the catastrophic illness subgroup and the other subgroup, respectively, both of which were ill-defined symptoms/signs. With the exception of ill-defined symptoms/signs, the four most frequent diagnoses for the normal population aged 65 years or older in Taiwan were found to be diseases of the circulation system, diseases of the respiratory system, injury/poisoning and diseases of the digestive system [26]. This was similar to the distribution of the identified diagnostic categories in the elderly with catastrophic illnesses at EDs found in the present study.

The present study is a descriptive epidemiological survey to evaluate the ED utilization of the elderly Taiwanese population, and some limitations exist. Possible sources of uncontrolled confounding in the present study, for example, social-economic influences, family support, complex co-morbidity or pharmacological management strategies, were not evaluated. Further investigations using data from a community-based interview survey will be performed.

Some limitations exist in the present study. First, a certificate of any catastrophic illness was confirmed based on the definitions announced by the Taiwan’s Health authority but according to the patients’ application. A misclassification bias could be happen when analyzing the present data because some lowly socio-ecological leveled or educated people didn’t know and use the welfare of the health insurance system. Second, the resent study was a cross-sectional study merely to describe the differences in EDs utilization and direct medical expenditure between the catastrophic and non-catastrophic illnesses. Much more interesting comparisons between the two groups, such as consequent lifespan, quality of life et al., could not be obtained. That needs other prospective surveys in future.

## Conclusions

In Taiwan, the elderly with an officially certificated catastrophic illness had significantly more ED visits and a higher ED medical cost; they also used greater emergency medical resources owing to different medical complaints as compared to those without a catastrophic illness.

## Competing interests

All authors declare that they have no conflicts of interest, including directorships, stock holding or contracts.

## Authors’ contributions

The study was designed by NPY and YHL; data were gathered and analyzed by CYC and JCH; the initial draft of the manuscript was written by NPY and ILY; and the accuracy of the data and analyses was assured by NTC and CLC. All authors participated in the preparation of the manuscript and approved the final version.

## Pre-publication history

The pre-publication history for this paper can be accessed here:

http://www.biomedcentral.com/1472-6963/13/152/prepub
